# Intersectoral Collaboration Between Traditional Bonesetters and Formal Healthcare: A Systematic Review on Past Initiatives and Stakeholder Perspectives

**DOI:** 10.1002/wjs.12503

**Published:** 2025-02-06

**Authors:** Joost J. Binnerts, Thom C. C. Hendriks, Samia Hussein, Nefti Bempong‐Ahun, Geoffrey C. Ibbotson, William J. Harrison, Claude Martin, Kavitha Ranganathan, Anam N. Ehsan, Bwire M. Chirangi, Michael J. R. Edwards, Erik Hermans

**Affiliations:** ^1^ Department of Surgery Radboud University Medical Centre Nijmegen The Netherlands; ^2^ Stichting Shirati Amsterdam The Netherlands; ^3^ Department of Surgery Slingeland Hospital Doetinchem The Netherlands; ^4^ Vrije Universiteit Medical Centre Amsterdam The Netherlands; ^5^ Global Surgery Foundation Geneva Switzerland; ^6^ AO Alliance Davos Switzerland; ^7^ Division of Plastic and Reconstructive Surgery Brigham and Women's Hospital Boston Massachusetts USA; ^8^ Shirati KMT Hospital Shirati Tanzania

**Keywords:** bonesetter, bonesetting, collaboration, fracture, integrative medicine, traditional medicine

## Abstract

**Background:**

Bone fractures in low‐ and middle‐income countries are commonly managed by traditional bonesetters (TBSs). Past studies emphasize the potential for improved fracture care through intersectoral cooperation. This review gauged support among stakeholders for intersectoral collaboration and the results of previous initiatives.

**Methods:**

Five medical databases were reviewed. Studies focusing on stakeholder perspectives and articles detailing collaborative initiatives were included. Data extraction and synthesis were carried out using the Cochrane Consumers and Communication Review Group's template. Additionally, all studies underwent quality assessment.

**Results:**

Of the 3821 identified articles, 16 were included after full‐text screening. Twelve articles presented stakeholder perspectives, whereas four discussed collaborative initiatives. The overall article quality was low: articles on stakeholder perspectives scored on average 1.42 out of 4 points, whereas articles on collaborative initiatives scored a mean 1.25 points. In total, 62% of stakeholders (75% of TBSs, 92% of hospital staff, and 52% of patients) expressed support for intersectoral collaboration. The ratio between stakeholders expressing support versus those opposing was 4.4:1. No articles presented data on governmental perspectives. The most mentioned collaborative forms were TBS training (24% of stakeholders) and an integrative model (16% of stakeholders). Interventional studies all consisted of TBS training, reporting improved clinical outcomes and increased practice integration.

**Conclusion:**

Despite the limited and low‐quality evidence on collaboration initiatives and perspectives, most stakeholders seem supportive of intersectoral collaboration, with training and integration being commonly suggested. Future research efforts exploring the feasibility of embedding TBSs into current primary care systems should ensure the involvement of local and national government.

## Graphical abstract

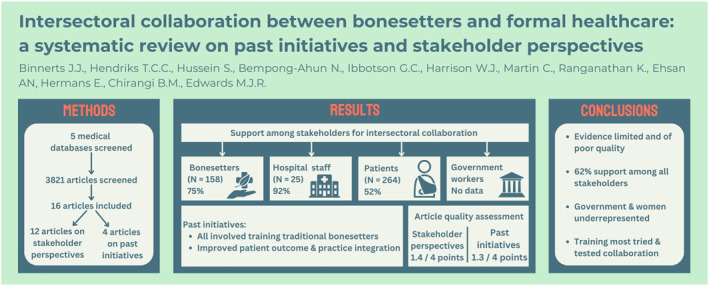

**Abbreviations**
CINAHL: Cumulative Index to Nursing and Allied Health Literature
LMIC: low‐ and middle‐income country
PRISMA: Preferred Reporting Items for Systematic Reviews and Meta‐Analyses
TBS: traditional bonesetter
WHO: World Health Organization
WoS: World of Science

## 1 Introduction

Musculoskeletal injuries and bone fractures are a global public health issue, causing a significant disease burden. According to the Global Burden of Disease Study 2019, the global incidence and prevalence of bone fractures in 2019 were 178 million and 455 million, respectively [1]. These injuries often result in lifelong disabilities, accounting for 25.8 million years lived with disability as of 2019 [1]. The impact extends beyond the individuals affected, placing a substantial strain on healthcare systems and communities through direct and indirect costs, including decreased productivity and significant economic losses.

In low‐ and middle‐income countries (LMICs), road traffic injuries are a major cause of bone fractures. The World Health Organization (WHO) reports that 93% of road traffic injury deaths occur in LMICs, with the African continent experiencing the highest rate of these fatalities [2]. Despite the significant burden of bone fractures resulting from these injuries, many sub‐Saharan African countries have a disproportionately small surgical workforce, hindering their ability to adequately address this issue. For instance, The United States count 9.2 orthopedic surgeons per 100,000 population [3], whereas Tanzania's surgical specialist workforce was estimated to be 0.46 per 100,000 population, with no data on the proportion of orthopedic specialists available [4].

When dealing with bone fractures, patients in rural areas of LMICs tend to prefer traditional bonesetters (TBSs) over formal healthcare. TBSs do not work in the regulated health field and often employ a combination of fracture splinting, massage and herbal ointments. Although relatively simple fractures may heal acceptably this way, patient with complex fractures are likely to develop serious disabilities such as mal‐ or nonunion, or osteomyelitis. Alternatively, formal healthcare is defined as structured systems that involve licensed professionals and institutions, which follow standardized practices and regulations. Reasons for patients to prefer TBSs include higher cultural acceptability, affordability, geographic convenience, and the limited availability of orthopedic trauma care. Earlier studies in rural Nigeria and Ghana showed that over 70% of fracture patients consult these traditional practitioners [5, 6]. Although these data suggest that TBSs could offer a partial solution in alleviating the burden on orthopedic surgical care, several other studies point out the complications associated with traditional bonesetting [7, 8, 9]. Currently, this has led to conflicting recommendations, with some authors calling for governmental mitigation of TBSs [10, 11], and others arguing for increased collaboration to reduce TBS‐related complications [12, 13]. However, there remains a significant gap in understanding the perspectives of various stakeholders—patients, traditional bonesetters, hospital staff, and government officials—regarding the appropriate approach to traditional bonesetting. Specifically, should there be collaboration between hospitals and TBSs, or should these practices remain distinct and be mitigated?

To our knowledge, there is currently no comprehensive review that critically assesses and summarizes the published literature on stakeholder perspectives in regard to professional cooperation between TBSs and the formal healthcare sector. The same applies to the literature available on any attempted collaborative initiatives between both parties. Therefore, the goal of this systematic review is to compile the current literature available on the perspectives of relevant stakeholders on intersectoral collaboration, to guide future policy on fracture care in rural areas.

## 2 Methods

### 2.1 Overview

We conducted a systematic review, exploring perspectives among stakeholders on intersectoral collaboration and previous initiatives toward this goal. This study did not require institutional review board approval because it used publicly available data sources and is a review article. The PRISMA (Preferred Reporting Items for Systematic reviews and Meta‐Analyses) reporting guidelines were used in the study's design [14].

### 2.2 Search Strategy and Selection Criteria

Five databases, PubMed, Embase, Web of Science (WoS), Cumulative Index to Nursing and Allied Health Literature (CINAHL), and Google Scholar were searched from database inception until July 2023. The search was repeated in September 2023, to ensure inclusion of the most recent literature. The following search terms (and their synonyms) were used: ‘traditional bonesetting’ and ‘traditional African medicine’, ‘extremity fracture’, ‘limb fracture’, and ‘bone fracture’ (see Supporting Information). To ensure robustness of the search strategy, information specialists of the Radboud UMC were involved in its development. Two types of studies were appraised:

– Articles exploring perspectives of TBSs, patients/community, hospital staff, and government employees on intersectoral collaboration.
– Articles studying interventions toward intersectoral collaboration

Original, full‐text, peer‐reviewed, articles in the English language were included. We used the WHO definition for intersectoral collaboration, which is “a recognized relationship between part or parts of the health sector with parts of another sector which has been formed to take action on an issue to achieve health outcomes (or intermediate health outcomes) in a way that is more effective, efficient or sustainable than could be achieved by the health sector acting alone” [15].

Two investigators (S.H. and J.B.) independently screened the titles and abstracts of the identified articles, with subsequent full‐text review of eligible articles using the web‐based version of Rayyan [16]. In case of disagreement between S.H. and J.B. during the discussion, a third investigator (T.H.) made the final selection. This methodology was applied to both the first and second search. Backward and forward snowballing was conducted using the screened full‐text articles to identify any additional relevant articles.

### 2.3 Data Analysis

The Cochrane Consumers and Communication Review Group's extraction template was used for data extraction [17]. After a pilot using three articles, the extraction sheet was reviewed by S.H., J.B., and T.H. and modified to ensure validity. Data extraction encompassed basic stakeholder demographics, study design, and support for intersectoral collaboration. Interventional studies toward intersectoral collaboration were assessed for effectiveness. Independent data extraction was carried out by S.H. and J.B., with T.H. as a third assessor in case of persisting variance.

Quantitative outcome measures included the proportion of respondents indicating support for intersectoral collaboration and suggested form of collaboration. To synthesize heterogeneous data, these forms of intersectoral collaboration were categorized into ‘provision of funds/infrastructure/equipment’, ‘training’, and ‘integrative model’. An integrative model was defined as any structural collaboration in the treatment of fracture patients, involving input from both sides.

A quality assessment tool of the Cochrane Collaboration Qualitative Methods Group was modified to include a quantitative grading system and allow comparison between studies (see Supporting Information) [18]. Articles on stakeholder perspectives were assigned 1 points each for credibility, transferability, dependability, and confirmability to a maximum score of 4. A score of 0–1 point was defined as ‘low quality’, 2–3 points as ‘medium quality’, and 4 points as ‘high quality’.

Interventional studies were similarly assessed for internal and external validity, reliability, and objectivity. Two investigators (S.H. and J.B.) performed the quality assessment individually, finding consensus afterward.

## 3 Results

### 3.1 Search Results

The first search, from database inception up to July 2023, identified 3629 records. After the removal of 913 duplicates, 2716 records underwent title and abstract screening. A total of 99 records underwent full‐text screening, and 14 records were included. Backward snowballing yielded one additional record.

**FIGURE 1:**
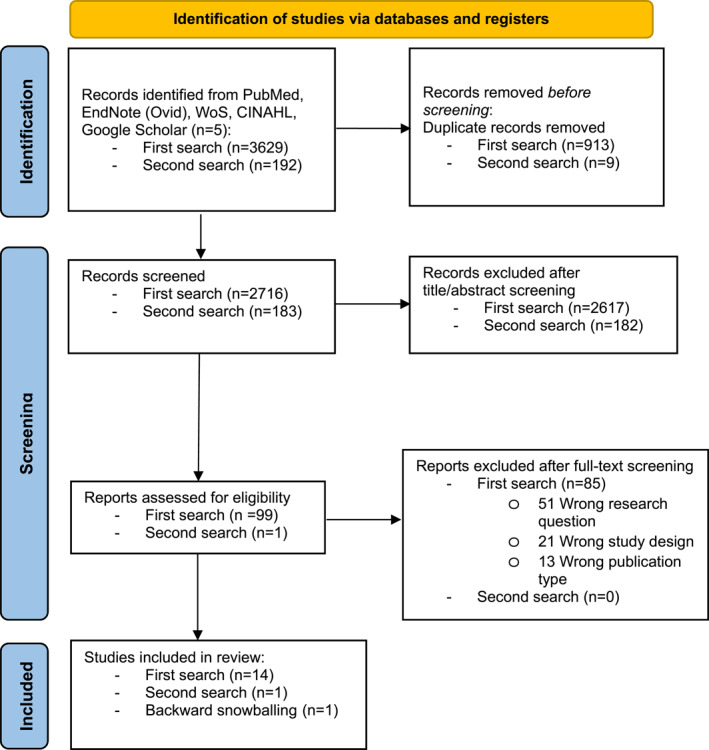
PRISMA flowchart for study selection: first and second search and forward snowballing.

The second search of the same databases (from database inception, up to September 2023) identified 192 new records. Nine duplicates were removed, and 183 records were screened. Out of this selection, one additional record was added. Thus, a total of 16 studies were included in this review (see Figure 1).

Twelve articles explored stakeholder perspectives on collaboration, totaling 447 participants, whereas four articles described interventional studies, involving a total of 637 participants (see Table 1). The majority of studies were carried out in Sub‐Saharan Africa (14/16 studies, 87.5%), where Nigeria was the most common setting (5/14 studies, 36%).

**TABLE 1:** Included study characteristics.

Article	Year	Country	Study type	Sample (*N*)
Yempabe et al. [6]	2020	Ghana	Explorative	28 TBSs
Onyemaechi et al. [19]	2020	Nigeria	Explorative	5 TBSs 8 hospital staff
Card et al. [13]	2020	Tanzania	Explorative	6 TBSs
Idris et al. [20]	2010	Sudan	Explorative	16 TBSs
Alegbeleye et al. [21]	2019	Cameroon	Explorative	5 TBSs 15 patients
Hancock et al. [22]	2022	Chad	Explorative	6 TBSs 2 hospital staff 25 patients
Udosen et al. [23]	2006	Nigeria	Explorative	6 TBSs
Isaacs‐Pullins et al. [24]	2022	India	Explorative	6 TBSs
Yempabe et al. [25]	2020	Ghana	Explorative	94 patients
Bassey et al. [26]	2009	Nigeria	Explorative	6 TBSs
Asa et al. [27]	2018	Nigeria	Explorative	69 TBSs 15 hospital staff 130 patients
Heinzerling [28]	2005	Cameroon	Explorative	5 TBSs
Shah et al. [29]	2003	Nepal	Training	367 village health workers
Eshete [30]	2005	Ethiopia	Training	112 TBSs
Onuminya [31]	2006	Nigeria	Training	1 TBS
Konadu‐Yeboah et al. [32]	2023	Ghana	Training	157 TBSs

### 3.2 Quality Assessment

Among the 12 studies on stakeholder perspectives, six studies were deemed as ‘low quality’, five studies ‘medium quality,’ and one study as ‘high quality’. The average quality score was 1.4 points (SD 1.4), often due to small sample sizes and/or significant exposure to bias. Among the interventional studies, two studies were deemed as ‘low quality’, one study as ‘medium quality’ and one was deemed as ‘high quality’. The average quality score of these studies was 1.3 points (SD 1.6) (see Table 2).

**TABLE 2:** Quality assessment of included articles.

Article	Credibility/internal validity	Transferability/external validity	Dependability/reliability	Confirmability/objectivity	Quality rating
Yempabe et al. [6]	—	+	—	—	1 (low)
Onyemaechi et al. [19]	+	+	+	—	3 (medium)
Card et al. [13]	+	+	+	+	4 (high)
Idris et al. [20]	—	—	—	—	0 (low)
Alegbeleye et al. [21]	—	—	—	—	0 (low)
Hancock et al. [22]	+	—	+	—	2 (medium)
Udosen et al. [23]	—	—	—	—	0 (low)
Isaacs‐Pullins et al. [24]	+	—	—	+	2 (medium)
Yempabe et al. [25]	—	+	—	+	2 (medium)
Bassey et al. [26]	—	—	—	—	0 (low)
Asa et al. [27]	+	—	+	+	3 (medium)
Heinzerling [28]	—	—	—	—	0 (low)
Shah et al. [29]	—	—	+	—	1 (medium)
Eshete [30]	—	—	—	—	0 (low)
Onuminya [31]	—	—	—	—	0 (low)
Konadu‐Yeboah et al. [32]	+	+	+	+	4 (high)

### 3.3 Articles on Stakeholder Perspectives

TBS perspectives on collaboration were reported in 11 studies, where a total of 158 TBSs were interviewed (see Table 3). Respondents were predominantly male, and displayed a diverse range of educational backgrounds, with the highest number having secondary schooling (28%), followed closely by primary education (23%) and no schooling (20%). Although age was heterogeneously reported, most TBSs seemed to be in the 40–60 years range. All reported having extensive experience in their field of practice, with a median of 17 years.

**TABLE 3:** Overview of TBS characteristics and perspectives.

Variable	Number (%)
Total respondents	158 (100%)
*Age*	Heterogeneous reporting (see Supporting Information)
*Sex*	
Male	139 (88%)
Female	14 (9%)
Not specified	5 (3%)
*Education*	
No formal education	32 (20%)
Primary education	36 (23%)
Secondary education	44 (28%)
Post‐secondary education	14 (9%)
Other education	11 (7%)
Not specified	21 (13%)
*Place of learning*	
Family	56 (35%)
Apprenticeship	16 (10%)
Divine calling	5 (3%)
Self‐education	1 (1%)
Not specified	80 (51%)
*Years of experience*	Mentioned in 7 articles
	Heterogeneous reporting (see Supporting Information)
*Support for collaboration*	
In favor of collaboration	118 (75%)
Not in favor of collaboration	34 (22%)
Not specified	6 (3%)
*Suggested form of collaboration (multiple responses possible)*	
Integrative model	67 (42%)
Training	95 (60%)
Provision of funds/infrastructure/equipment	50 (32%)

A majority of TBS respondents across all studies (75%) expressed their support for intersectoral collaboration. The most frequently mentioned facilitators to such a partnership among TBSs were respect and equality, cited in four (36%) of the 11 articles.

When asked about the best way to shape a cooperation between TBSs and formal healthcare, three main themes emerged. Firstly, TBS training appeared most popular, being mentioned in 9 out of 11 articles (82%), and supported by 60% of all TBS respondents. In the study of Onyemaechi et al, one TBS said the following: *“There's an idea from science that we may not know, a time can come for a seminar to be organized for us or it can be in a class where a professor can come and teach. There are some machines hospitals use that we may need. We can only be trained on how to handle them, like testing machine. So those things, we may not have personal experience of how to handle them but we can be taught”.* Other suggested topics for training included management of complications, basic fracture management, and record‐keeping.

Secondly, an integrative model was mentioned in 9 out of 11 articles (82%), and was favored by 42% of TBS respondents. Such a model could have several forms, in the study of Card et al., one TBS said: *“The best way is coming together, working together, and identifying shortages in hospital procedures and traditional procedures”.* Another TBS in this study suggested pairing him with a hospital healthcare provider, who could administer anesthesia during fracture manipulation by the TBS.

Lastly, provision of equipment or funds to provide better fracture care was proposed by 32% of all TBS respondents and reported in 3 out of 11 (27%) studies.

Three studies reported on the perspectives of healthcare professionals regarding a collaboration with traditional bonesetters, comprising a total of 25 respondents. In general, orthopedic surgeons were interviewed (specialists 72% vs. residents 20%), of whom most were male (96% vs. 4% female). Both age and years of experience were categorized differently, but suggested that most staff were between 30 and 40 years old and had at least 5 years of experience (see Table 4).

**TABLE 4:** Overview of hospital staff characteristics and perspectives.

Variable	Number (%)
Total respondents	25 (100%)
*Age*	Heterogeneous reporting (see Supporting Information)
*Sex*	
Male	24 (96%)
Female	1 (4%)
*Education*	
Orthopedic specialist	18 (72%)
Surgical resident	5 (20%)
Not specified	2 (8%)
*Support for collaboration*	
In favor of collaboration	23 (92%)
Not in favor of collaboration	2 (8%)
*Suggested form of collaboration (multiple responses possible)*	
Integrative model	6 (24%)
Training	10 (40%)
Unspecified	15 (60%)

Intersectoral collaboration was supported by 92% of orthopedic surgeons interviewed. Their preferred method to achieve this was through training of TBSs (40% of respondents, mentioned in the two articles that posed this question to respondents).

In the study by Onyemaechi et al., one orthopedic surgeon commented the following: *“…my area of emphasis is on patient or client selection. It will go a long way to limit the damage. If we are able to get that one right, then we can step up to the things they should do during their practice, but patient selection is the first, I think that is where the friendliness should start. If we start by telling them what to do in their practice, they will tell you that their inspiration comes from the spirit”.*

An integrative model was only mentioned in the article by Onyemaechi et al., in which 6 out of 8 surgeons (75%) supported this notion. One of the participating surgeons shared his view on implementation: *“Integrating them into the primary healthcare system will be a good idea considering their huge patronage. Instead of making them illegal and they remain in the dark, causing havoc, it is better they are given a legal platform”.* Legislation was seen as crucial to successful collaboration by hospital staff in both the study by Onyemaechi et al. and Hancock et al.

Provision of funds/infrastructure/equipment for traditional bonesetters was not proposed by hospital staff within these studies.

Four out of the 12 explorative studies contained information about patient/community views on collaboration, seeing a total of 264 patients/community members. The majority of the interviewees were male (69% vs. 31% female), aged around 30–40 years old, with a broad range of educational level (see Table 5).

**TABLE 5:** Overview of patient/community characteristics and perspectives.

Variable	Number (%)
Total respondents	264 (100%)
*Age*	Heterogeneous reporting (see Supporting Information)
*Sex*	
Male	182 (69%)
Female	82 (31%)
*Education*	
No formal education	30 (11%)
Primary education	39 (15%)
Secondary education	16 (6%)
Post‐secondary education	1 (0.4%)
Other education	12 (4%)
Not specified	166 (63%)
*Support for collaboration*	
In favor of collaboration	138 (52%)
Not in favor of collaboration	27 (10%)
Not specified	99 (38%)
*Suggested form of collaboration (multiple responses possible)*	
Integrative model	3 (1%)
Training	2 (1%)
Not specified	259 (98%)

Out of the responses specified in the four studies, a majority indicated support for intersectoral collaboration (52% in favor vs. 10% opposed and 38% not reported). Yempabe et al. stated that, in their focus group with 30 patients, “Many participants indicated that they would want orthodox doctors to help TBSs with pain management because bonesetting procedures can be very painful. They also want doctors to help TBSs manage open fractures and to train them to read x‐rays”. No dissenting opinions were noted in this focus group.

In the study by Hancock et al., one community member said: “*The community needs both Jabari (Chadian TBSs) and doctors; they each are good at their specialty. To improve healthcare in Am Timan (city in Chad), they need more specialists like Jabari and doctors to train other people*”.

No studies reported on the perspectives of government officials on intersectoral collaboration.

Synthesis of all stakeholder perspectives (*N* = 447) from a total of 12 studies yielded 62% (*N* = 279) of respondents supporting intersectoral collaboration, 14% (*N* = 63) respondents opposing this and 24% (*N* = 105) whose views were unspecified. The ratio between respondents supporting collaboration versus those opposed is 4.4 to 1.

Concerning the preferred form of cooperation, 24% (*N* = 107) of all stakeholders favored TBS training, followed by 16% (*N* = 73), supporting an integrative model. Provision of funds/infrastructure/equipment was proposed by 50 stakeholders (11%), all of whom were TBSs.

### 3.4 Interventional Studies

All four interventional studies entailed training of TBSs, health assistants, and/or community health workers. Training duration varied from one to five days. All studies included a follow‐up period, with a median duration of 24 months (range 6–72 months); the median follow‐up rate was 41% (range 35%–100%). Sample sizes differed strongly between the studies, ranging from 1 to 367 participants (see Table 6).

**TABLE 6:** Interventional studies on TBS training.

Study	Intervention design	Duration (days)	Number of participants (*N*)	Follow‐up duration (months)	Follow‐up rate (%)	Training content	Study outcomes			
Eshete	2‐year retrospective analysis versus 2‐year prospective analysis	3	112	24	44	– Not specified	– 25 amputations versus 49 pre‐training – 7 TBS‐related gangrene versus 25 pre‐training – 2 deaths versus 3 pre‐training			
Onuminya	Trained Center A versus untrained Center B	1	1	24	100	– Conservative fracture treatment indications – Patient selection – Basic principles of fracture treatment – Prevention of complications – Referral services and outcome of treatment		Intervention		Control
							Acceptable union	19 (48%)		5 (13%)
							Delayed union	7 (18%)		8 (20%)
							Malunion	8 (20%)		12 (30%)
							Nonunion	3 (8%)		6 (15%)
							Osteomyelitis	2 (5%)		5 (13%)
							Limb gangrene	1 (3%)		4 (10%)
							Total	40 (100%)		40 (100%)
Shah et al.	Training with 6‐monthly follow‐up	5	367	72	35	– Primary care of common injuries, wounds, and complications – Referring/follow‐up of patients – Bandaging, splinting and plaster techniques	Mean pre‐test score: 6.47 out of 10 Mean post‐test score: 8.67 out of 10 Paired *t*‐test: *p* > 0.05			
Konadu‐Yeboah et al.	Training	4	157	6	38	– Biology of fracture‐healing – Fracture diagnosis without X‐ray imaging – Complications of fractures – Joint dislocations and treatment – Fracture bandaging – Medicolegal implications of TBS practice		Pre‐test (out of 5)	Post‐test (out of 5)	*p*‐value
							Nature of bone and soft tissues	3.62	4.24	< 0.0001
							Fractures in children	3.64	4.38	< 0.0001
							Splinting and bandaging	5.57	8.04	< 0.0001
							Hand hygiene	2.43	2.42	0.855
							Fracture complications	2.01	2.82	< 0.0001
							Pain management	2.77	3.61	< 0.0001
							Fracture healing	6.68	8.18	< 0.0001
							Record‐keeping	0.49	0.73	0.028
							37 TBS referrals in 6 months post‐training			

All interventional studies reported an improvement in the measured outcomes after training. The study by Konadu‐Yeboah et al. was considered as a high quality study, in which 157 TBSs received training during 4 days by a team of orthopedic specialists. Significantly improved post‐training test scores were seen across all areas, except hand hygiene, with an overall knowledge gain of 19.7 percentage points compared to baseline. A 6‐month follow‐up showed partial knowledge retention. Furthermore, the study demonstrated that trained TBSs had referred 37 fracture cases to a local hospital. However, pre‐training referral numbers were unavailable.

Eshete reported a nearly 50% reduction in amputations with concordant decrease in TBS‐related gangrene during the 2‐year prospective period following the training, compared to the 2‐year retrospective cohort pre‐training. An unspecified number of health assistants also received training; therefore it remains uncertain what proportion of this reduction can be attributed to training TBSs.

Onuminya found higher numbers of adequate union in tibial fracture patients treated by a trained TBS in Center A, as compared to tibial fracture patients treated by an untrained TBS in Center B. It should be noted that no data are available prior to the training. Furthermore, in both the study by Eshete and Onuminya, no statistical analysis was performed to demonstrate a significant difference in outcomes.

Shah et al. described significantly higher knowledge test scores post‐training, as well as sustainable changes in the practices of the trainees. There was a demonstration of better fracture management, and, consequently, improved patient safety. However, the degree of prior medical education of the participants, who are described as village health workers, was not clearly noted.

## 4 Discussion

This is the first review that critically assessed the literature on potential benefits and challenges of intersectoral collaboration between traditional bonesetters and formal healthcare systems. It was demonstrated that a majority (62%) of TBSs, hospital staff, and patients support increased cooperation, with TBS training (24%) and an integrative model (16%) most commonly proposed. The four interventional studies included in this review all involved TBS training, suggesting beneficial effects on trainee knowledge and skills, as well as improved patient outcomes.

In this study, hospital staff respondents demonstrated the highest level of enthusiasm for collaboration, with 92% in favor, compared to 75% of traditional bonesetters and 52% of patients and community members. Despite representing a relatively small stakeholder group, this finding contradicts the hypothesis that greater exposure to TBS‐related complications would render hospital staff more opposed to collaboration. However, it correlates well with a recently published consensus statement, in which orthopedic surgeons from several African nations advocated for the registration of TBS‐related complications, as well as training TBSs to reduce these complications and allowing them to be embedded into formal healthcare [33].

Although patients and community members exhibited the lowest percentage of support for collaboration, a significant proportion of individuals in this group held an unspecified stance. The ratio of supporters to opponents among patients and community members was 5.1:1, exceeding the overall stakeholder support‐to‐opposition ratio of 4.4:1. This emphasizes the supportive views of the most important stakeholder group, namely, the beneficiaries of such collaborative fracture care. No consistent changes in stakeholder opinions over time were noted across the included studies.

This review identified two significant gaps in stakeholder perspectives, resulting in an incomplete understanding of this research area: firstly, none of the included studies provided data on the perspectives of government officials. Considering the crucial role of governmental policies and regulatory frameworks in facilitating and sustaining intersectoral collaboration, this omission represents a significant blind spot.

Secondly, a significant sex disparity was observed in the included studies, predominantly favoring males across all stakeholder groups. Although traditional bonesetting is typically a male‐dominated occupation in certain regions, as noted by Hancock et al. [22], female bonesetters are active in various other countries [19, 20, 21]. Furthermore, disability‐adjusted life years due to fractures have increased more for females than males from 1990 to 2019, primarily due to osteoporosis among elderly women [34]. Lastly, the surgical workforce in LMICs is increasingly addressing the underrepresentation of women through targeted scholarship programs [35]. Therefore, it is imperative to ensure adequate representation of women in all stakeholder groups in future explorative studies.

The most commonly suggested form of collaboration was TBS training. This was a consistent theme among all the interventional studies in this review. These initiatives suggested that such collaborations can lead to improved clinical outcomes and better integration of TBSs into the healthcare system. However, the quality of the evidence is a concern, with most studies being of low quality due to poorly described methodology, highlighting the need for more rigorous research in this area. A consensus meeting prior to the training, involving both trainers and trainees, has been suggested to enhance fracture care education [36]. An integrative model was mentioned by 73 stakeholders (16%), although no specific initiatives have been described in detail thus far. Omololu et al. have proposed a triage and referral algorithm, in which relatively simple fractures are treated traditionally, and more complicated fractures are referred to the local hospital [37]. However, such a model is yet to be tested and would still leave a separation between traditional bonesetters and the formal healthcare sector. To bridge this, it may be necessary to form local ‘collaborative fracture management teams’, which could discuss fracture cases to see what each side might contribute to the management. Other studies support this idea, emphasizing the importance of a ‘third party organization’, which could act as a bridge builder, legislator, and a mediator [5, 12].

This study has important limitations, including a language bias, as the investigators were only fluent in Dutch, English, and Kiswahili. Since China has a rich history of traditional bonesetting and advanced integration within its formal healthcare sector, the inclusion of Chinese articles could have provided valuable input. However, a wide range of international studies were still captured. There was also a lack of prospective notification at a registry, such as PROSPERO. Retrospectively, PROSPERO was investigated for articles of a similar nature and none were found to be registered. Finally, a notable limitation in this body of research was the inconsistency in data reporting among included studies, which generally lacked individual participant data. This inconsistency introduces the risk of data interpretation not reflecting the underlying reality.

## 5 Conclusions

Based on this present review of the current literature, intersectoral collaboration between traditional bonesetters and formal healthcare systems is generally supported and holds promise for improving primary orthopedic trauma care in LMICs. However, this evidence base is limited by the poor quality of available studies and the lack of data on certain stakeholder groups. Future research should aim to address these gaps by conducting high‐quality, rigorous studies that assess the effectiveness, sustainability, and scalability of intersectoral collaboration. Involvement of government officials and policymakers in this research is crucial to ensure that findings can be translated into practice and supported by appropriate policy frameworks.

Ultimately, the goal of intersectoral collaboration should be to leverage the strengths of both traditional and formal healthcare systems. Doing so could mitigate each sector's weaknesses and create a more integrated, inclusive, and effective primary trauma care system that meets the needs of all individual patients, particularly those in underserved and rural areas.

## Author Contributions

**Joost J. Binnerts**: conceptualization, data curation, formal analysis, funding acquisition, investigation, methodology, project administration, resources, supervision, validation, visualization, writing–original draft, writing–review and editing. **Thom C. C. Hendriks**: conceptualization, data curation, formal analysis, investigation, methodology, project administration, supervision, writing–review and editing. **Samia Hussein**: data curation, formal analysis, investigation, methodology, project administration, writing–original draft. **Nefti Bempong‐Ahun**: project administration, resources, supervision, writing–review and editing. **Geoffrey C. Ibbotson**: project administration, resources, writing–review and editing. **William J. Harrison**: project administration, supervision, writing–review and editing. **Claude Martin**: project administration, supervision, writing–review and editing. **Kavitha Ranganathan**: supervision, validation, writing–review and editing. **Anam N. Ehsan**: supervision, validation, writing–review and editing. **Bwire M. Chirangi**: project administration, resources, supervision, writing–review and editing. **Michael J. R. Edwards**: resources, supervision, validation, writing–review and editing. **Erik Hermans**: conceptualization, project administration, resources, supervision, validation, writing–review and editing.

## Ethics Statement

The authors have nothing to report.

## Consent

The authors have nothing to report.

## Conflicts of Interest

The authors declare no conflicts of interest.

## Supporting information

Supporting Information S1

Supporting Information S2

Supporting Information S3

Supporting Information S4

Supporting Information S5

## Data Availability

All data generated or analyzed during this study are included in this published article and its supplementary information files.
