# Supplementary material for: Intersectoral Collaboration Between Traditional Bonesetters and Formal Healthcare: A Systematic Review on Past Initiatives and Stakeholder Perspectives
Source: World J Surg. 2025 Feb 6;49(3):652–63. doi: 10.1002/wjs.12503 (PMC11903250; doi:10.1002/wjs.12503)
Supplement: Supplementary file 2 — Supporting Information S2 [file WJS-49-652-s005.docx]

Appendix B: Search strategy

# First search: 3629 results

Table D.1: First search strategy in PubMed

| PubMed (25-07-2023) | | |
| --- | --- | --- |
| Search number | Query | Results |
| *1* | bone sett*[tiab] OR bonesett*[tiab] OR bone healer*[tiab]  OR  (("medicine, african traditional"[MeSH] OR "traditional medicine practitioners"[Mesh] OR "health services, indigenous"[Mesh] OR traditional medicin*[tiab] OR indigenous medicin*[tiab] OR ethnomedicine*[tiab] OR folk remed*[tiab])  AND  ("fractures, bone"[MeSH] OR "fracture healing"[Mesh] OR ("bone and bones"[MeSH] OR bone*[tiab] OR extremit*[tiab] OR limb*[tiab] OR leg[tiab] OR legs[tiab] OR arm[tiab] OR arms[tiab] OR elbow*[tiab] OR ankle*[tiab]) AND (fracture*[tiab] OR broken[tiab] OR break*[tiab])))) | *423* |
| *URL* | https://pubmed.ncbi.nlm.nih.gov/?term=bone+sett%2A%5Btiab%5D+OR+bonesett%2A%5Btiab%5D+OR+bone+healer%2A+%5Btiab%5D+OR+%28%28%22medicine%2C+african+traditional%22%5BMeSH%5D+OR+%22traditional+medicine+practitioners%22%5BMesh%5D+OR+%22health+services%2C+indigenous%22%5BMesh%5D+OR+traditional+medicin%2A%5Btiab%5D+OR+indigenous+medicin%2A%5Btiab%5D+OR+ethnomedicine%2A%5Btiab%5D+OR+folk+remed%2A%5Btiab%5D%29+AND+%28%22fractures%2C+bone%22%5BMeSH%5D+OR+%22fracture+healing%22%5BMesh%5D+OR+%28%28%22bone+and+bones%22%5BMeSH%5D+OR+bone%2A%5Btiab%5D+OR+extremit%2A%5Btiab%5D+OR+limb%2A%5Btiab%5D+OR+leg%5Btiab%5D+OR+legs%5Btiab%5D+OR+arm%5Btiab%5D+OR+arms%5Btiab%5D+OR+elbow%2A%5Btiab%5D+OR+ankle%2A%5Btiab%5D%29+AND+%28fracture%2A%5Btiab%5D+OR+broken%5Btiab%5D+OR+break%2A%5Btiab%5D%29%29%29%29&ac=no&sort=relevance | |

Table D.2: First search strategy in Embase

| Embase (OVID) (25-07-2023) | | |
| --- | --- | --- |
| Search number | Query | Results |
| *1* | (bone sett* or bonesett* or bonehealer).ti,ab,kf. | *357* |
| *2* | (African medicine/ or traditional healer/ or indigenous health care/ or (traditional medicin* or indigenous medicin* or ethnomedicine* or folk remed*).ti,ab,kf.) and ((exp bone/ and exp fracture/) or ((limb* or bone* or extremit* or leg or legs or arm or arms or elbow* or ankle*) and (fracture* or broken or break*)).ti,ab,kf.) | *141* |
| *3* | 1 or 2 | *483* |
| *URL* | <https://ovidsp.ovid.com/ovidweb.cgi?T=JS&NEWS=N&PAGE=main&SHAREDSEARCHID=7C0YQf0FYzp90VMqtVFJcWdjMaF7zKhgUH2IWxxQHvspzjotYdw44l5cpLxJvj47i> | |

Table D.3: First search strategy in Web of Science

| Web of Science (26-07-2023) | | |
| --- | --- | --- |
| Search number | Query | Results |
| *1* | TS= (“bone sett*” OR bonesett* OR bone healer*) OR TS=((limb OR limbs OR bone OR bones OR extremity OR extremities OR leg OR legs OR arm OR arms OR elbow OR elbows OR ankle OR ankles) AND (fracture* OR broken) AND (traditional OR indigenous OR ethnomedicine* OR folk)) and Orthopedics (Web of Science Categories) | *788* |
| *URL* | [**https://www.webofscience.com/wos/woscc/summary/b01c17bc-8c57-49fa-ad86-af78b5bdcc13-990cfcd4/relevance/1**](https://www.webofscience.com/wos/woscc/summary/b01c17bc-8c57-49fa-ad86-af78b5bdcc13-990cfcd4/relevance/1) | |

Table D.4: First search strategy in CINAHL

| CINAHL (26-07-2023) | | |
| --- | --- | --- |
| Search number | Query | Results |
| *1* | TX bone-sett* OR bonesett* OR bone healer* | 92 |
| *2* | MH "Medicine, African Traditional" OR MH "Traditional Healers" | 1,751 |
| *3* | MH "Fractures+" | 68,217 |
| *4* | (MH "Bone and Bones+") | 148,865 |
| *5* | TX limb OR limbs OR bone OR bones OR extremities OR leg OR legs OR arm OR arms OR elbow OR elbows OR ankle OR ankles | 376,297 |
| *6* | TX fracture* OR broken OR break* | 150,548 |
| *7* | TX traditional OR indigenous OR ethnomedicine* OR folk remed* | 178,985 |
| *8* | S3 OR S6 | 150,951 |
| *9* | S4 OR S5 | 457,174 |
| *10* | S2 OR S7 | 178,985 |
| *11* | S8 AND S9 AND S10 | 1,414 |
| *12* | S1 OR S11 | 1,487 |
| *URL* | <https://search.ebscohost.com/login.aspx?direct=true&db=cin20&bquery=(TX+(bone-sett*+OR+bonesett*+OR+bone+healer*))+OR+(((MH+%26quot%3bFractures%2b%26quot%3b)+OR+(TX+fracture*+OR+broken+OR+break*))+AND+(((MH+%26quot%3bBone+and+Bones%2b%26quot%3b))+OR+(TX+limb+OR+limbs+OR+bone+OR+bones+OR+extremities+OR+leg+OR+legs+OR+arm+OR+arms+OR+elbow+OR+elbows+OR+ankle+OR+ankles))+AND+((MH+%26quot%3bMedicine%2c+African+Traditional%26quot%3b+OR+MH+%26quot%3bTraditional+Healers%26quot%3b)+OR+(TX+traditional+OR+indigenous+OR+ethnomedicine*+OR+folk+remed*)))&type=1&searchMode=Standard&site=ehost-live> | |

Table D.5: First search strategy in Google Scholar

| Google Scholar (26-07-2023) | | |
| --- | --- | --- |
| Search number | Query | Results |
| *1* | ‘traditional bone setter”\|’traditional bone setting”\|traditional bonesetter”\|traditional bonesetting”\|traditional bone setters"\|"traditional bonesetters" | *457* |

# Second search: 192 new results

Table D.6: Second search strategy in PubMed

| PubMed (20-09-2023) | | |
| --- | --- | --- |
| Search number | Query | Results |
| *1* | bone sett*[tiab] OR bonesett*[tiab] OR bone healer*[tiab]  OR  (("medicine, african traditional"[MeSH] OR "traditional medicine practitioners"[Mesh] OR "health services, indigenous"[Mesh] OR traditional medicin*[tiab] OR indigenous medicin*[tiab] OR ethnomedicine*[tiab] OR folk remed*[tiab])  AND  ("fractures, bone"[MeSH] OR "fracture healing"[Mesh] OR ("bone and bones"[MeSH] OR bone*[tiab] OR extremit*[tiab] OR limb*[tiab] OR leg[tiab] OR legs[tiab] OR arm[tiab] OR arms[tiab] OR elbow*[tiab] OR ankle*[tiab]) AND (fracture*[tiab] OR broken[tiab] OR break*[tiab])))) | *432* |
| *2* | #1 AND ("2023/07/15"[CRDT] : "3000"[CRDT] OR "2023/07/15"[EDAT] : "3000"[EDAT] OR "2023/07/15"[MHDA] : "3000"[MHDA]) | *9* |
| *URL* | https://pubmed.ncbi.nlm.nih.gov/?term=bone+sett%2A%5Btiab%5D+OR+bonesett%2A%5Btiab%5D+OR+bone+healer%2A+%5Btiab%5D+OR+%28%28%22medicine%2C+african+traditional%22%5BMeSH%5D+OR+%22traditional+medicine+practitioners%22%5BMesh%5D+OR+%22health+services%2C+indigenous%22%5BMesh%5D+OR+traditional+medicin%2A%5Btiab%5D+OR+indigenous+medicin%2A%5Btiab%5D+OR+ethnomedicine%2A%5Btiab%5D+OR+folk+remed%2A%5Btiab%5D%29+AND+%28%22fractures%2C+bone%22%5BMeSH%5D+OR+%22fracture+healing%22%5BMesh%5D+OR+%28%28%22bone+and+bones%22%5BMeSH%5D+OR+bone%2A%5Btiab%5D+OR+extremit%2A%5Btiab%5D+OR+limb%2A%5Btiab%5D+OR+leg%5Btiab%5D+OR+legs%5Btiab%5D+OR+arm%5Btiab%5D+OR+arms%5Btiab%5D+OR+elbow%2A%5Btiab%5D+OR+ankle%2A%5Btiab%5D%29+AND+%28fracture%2A%5Btiab%5D+OR+broken%5Btiab%5D+OR+break%2A%5Btiab%5D%29%29%29%29&ac=no&sort=relevance | |

Table D.7: Second search strategy in Embase

| Embase (OVID) (20-09-2023) | | |
| --- | --- | --- |
| Search number | Query | Results |
| *1* | (bone sett* or bonesett* or bonehealer).ti,ab,kf. | *362* |
| *2* | (African medicine/ or traditional healer/ or indigenous health care/ or (traditional medicin* or indigenous medicin* or ethnomedicine* or folk remed*).ti,ab,kf.) and ((exp bone/ and exp fracture/) or ((limb* or bone* or extremit* or leg or legs or arm or arms or elbow* or ankle*) and (fracture* or broken or break*)).ti,ab,kf.) | *144* |
| *3* | 1 or 2 | *491* |
| *4* | limit 3 to dc=20230715-20230930 | *9* |
| *URL* | <https://ovidsp.ovid.com/ovidweb.cgi?T=JS&NEWS=N&PAGE=main&SHAREDSEARCHID=2FRNwnKlY8EDsCCbbN2GbrVVSU4KBSAhbc5b1ZSl0AbDIzmFOAtpXZ3WeU2r08S5r> | |

Table D.8: Second search strategy in Web of Science

| Web of Science (20-09-2023) | | |
| --- | --- | --- |
| Search number | Query | Results |
| *1* | TS= (“bone sett*” OR bonesett* OR bone healer*) OR TS=((limb OR limbs OR bone OR bones OR extremity OR extremities OR leg OR legs OR arm OR arms OR elbow OR elbows OR ankle OR ankles) AND (fracture* OR broken) AND (traditional OR indigenous OR ethnomedicine* OR folk)) and Orthopedics (Web of Science Categories) | *804* |
| *2* | #1 Selected index date 2023-07-15 to 2023-09-30 | *17* |
| *URL* | <https://www.webofscience.com/wos/woscc/summary/2f6bfb86-ce3d-4fe4-9c30-5bed575be6a5-a54e1797/relevance/1> | |

Table D.9: Second search strategy in CINAHL

| CINAHL (21-09-2023) | | |
| --- | --- | --- |
| Search number | Query | Results |
| *1* | S1 AND S2 | 137 |
| *2* | EM 20230715- OR ZD "in process" | 948,706 |
| *3* | (TX (bone-sett* OR bonesett* OR bone healer*)) OR (((MH "Fractures+") OR (TX fracture* OR broken OR break*)) AND (((MH "Bone and Bones+")) OR (TX limb OR limbs OR bone OR bones OR extremities OR leg OR legs OR arm OR arms OR elbow OR elbows OR ankle OR ankles)) AND ((MH "Medicine, African Traditional" OR MH "Traditional Healers") OR (TX traditional OR indigenous OR ethnomedicine* OR folk remed*))) | 1,508 |
| *URL* | <https://search.ebscohost.com/login.aspx?direct=true&db=cin20&bquery=((TX+(bone-sett*+OR+bonesett*+OR+bone+healer*))+OR+(((MH+%26quot%3bFractures%2b%26quot%3b)+OR+(TX+fracture*+OR+broken+OR+break*))+AND+(((MH+%26quot%3bBone+and+Bones%2b%26quot%3b))+OR+(TX+limb+OR+limbs+OR+bone+OR+bones+OR+extremities+OR+leg+OR+legs+OR+arm+OR+arms+OR+elbow+OR+elbows+OR+ankle+OR+ankles))+AND+((MH+%26quot%3bMedicine%2c+African+Traditional%26quot%3b+OR+MH+%26quot%3bTraditional+Healers%26quot%3b)+OR+(TX+traditional+OR+indigenous+OR+ethnomedicine*+OR+folk+remed*))))+AND+(EM+20230715-+OR+ZD+%26quot%3bin+process%26quot%3b)&type=1&searchMode=Standard&site=ehost-live> | |

Table D.10: Second search strategy in Google Scholar

| Google Scholar (21-09-2023) | | |
| --- | --- | --- |
| Search number | Query | Results |
| *1 (customised data range: 2023-2023)* | ‘traditional bone setter”\|’traditional bone setting”\|traditional bonesetter”\|traditional bonesetting”\|traditional bone setters"\|"traditional bonesetters" | *20* |
