# Supplementary material for: Intersectoral Collaboration Between Traditional Bonesetters and Formal Healthcare: A Systematic Review on Past Initiatives and Stakeholder Perspectives
Source: World J Surg. 2025 Feb 6;49(3):652–63. doi: 10.1002/wjs.12503 (PMC11903250; doi:10.1002/wjs.12503)
Supplement: Supplementary file 3 — Supporting Information S3 [file WJS-49-652-s001.docx]

Appendix C: Eligibility criteria

**Table B.1**

| **Inclusion criteria** | **Exclusion criteria** |
| --- | --- |
| Qualitative studies, mixed-methods studies | Review articles |
| Interventional studies | Studies with sole focus on complications or TBS practice |
| Setting: low- or middle-income country (as defined by the World Bank 2021 list) | Setting: high-income country (as defined by the World Bank 2021 list) |
| Peer-reviewed studies | Study theses |
| Date range: database inception - September 2023 | Publication before January 1980 |
|  | Any language other than English |
