# Supplementary material for: Intersectoral Collaboration Between Traditional Bonesetters and Formal Healthcare: A Systematic Review on Past Initiatives and Stakeholder Perspectives
Source: World J Surg. 2025 Feb 6;49(3):652–63. doi: 10.1002/wjs.12503 (PMC11903250; doi:10.1002/wjs.12503)
Supplement: Supplementary file 4 — Supporting Information S4 [file WJS-49-652-s003.docx]

Appendix D: Quality Assessment

Qualitative studies

| *Study/Criterion* | Study 1 | Study 2 | Study 3 | Study 4 | Study 5 |
| --- | --- | --- | --- | --- | --- |
| Credibility |  |  |  |  |  |
| Transferability |  |  |  |  |  |
| Dependability |  |  |  |  |  |
| Confirmability |  |  |  |  |  |
| Quality rating |  |  |  |  |  |
| Comments |  |  |  |  |  |

- Quantitative studies

| *Study/Criterion* | Study 1 | Study 2 | Study 3 | Study 4 | Study 5 |
| --- | --- | --- | --- | --- | --- |
| Internal validity |  |  |  |  |  |
| External validity/Generalisability |  |  |  |  |  |
| Reliability |  |  |  |  |  |
| Objectivity |  |  |  |  |  |
| Quality rating |  |  |  |  |  |
| Comments |  |  |  |  |  |

- Quality rating

0-2 criteria met: Low

3 criteria met: Midden

4 criteria met: High

- For qualitative studies, the following criterions’ definitions have been used, as used in: Hannes K. Chapter 4: Critical appraisal of qualitative research. In: Noyes J, Booth A, Hannes K, Harden A, Harris J, Lewin S, Lockwood C (editors), *Supplementary Guidance for Inclusion of Qualitative Research in Cochrane Systematic Reviews of Interventions.* Version 1 (updated August 2011). Cochrane Collaboration Qualitative Methods Group, 2011. Available from URL <http://cqrmg.cochrane.org/supplemental-handbook-guidance>**:**
- **Credibility**: *Credibility* evaluates whether or not the representation of data fits the views of the participants studied, whether the findings hold true.
  Evaluation techniques include: having outside auditors or participants validate findings (member checks), peer debriefing, attention to negative cases, independent analysis of data by more than one researcher, verbatim quotes, persistent observation etc.
- **Transferability**: *Transferability* evaluates whether research findings are transferable to other specific settings.
  Evaluation techniques include: providing details of the study participants to enable readers to evaluate for which target groups the study provides valuable information, providing contextual background information, demographics, the provision of thick description about both the sending and the receiving context etc.
- **Dependability**: *Dependability* evaluates whether the process of research is logical, traceable and clearly documented, particularly on the methods chosen and the decisions made by the researchers.
  Evaluation techniques include: peer review, debriefing, audit trails, triangulation in the context of the use of different methodological approaches to look at the topic of research, reflexivity to keep a self-critical account of the research process, calculation of inter-rater agreements etc.
- **Confirmability**: *Confirmability* evaluates the extent to which findings are qualitatively confirmable through the analysis being grounded in the data and through examination of the audit trail.
  Evaluation techniques include: assessing the effects of the researcher during all steps of the research process, reflexivity, providing background information on the researcher’s background, education, perspective, school of thought etc.
- For quantitative studies, the following criterions’ definitions have been used (see below for references):
- **Internal validity:** Internal validity describes the extent of systematic error inherent to an individual study and reflects the extent to which the study’s methods can provide an unbiased result. Internal validity is assessed as the *risk of bias* ^1^
- **External validity/generalisability:** External validity describes the extent of systematic error in applying the results of a study to answer a specific question, which may also be referred to as “generalisability”, “applicability” or “directness” ^1^
- **Reliability:** Reliability refers to whether or not you get the same answer by using an instrument to measure something more than once. In simple terms, research reliability is the degree to which a research method produces stable and consistent results. A specific measure is considered to be reliable if its application on the same object of measurement number of times produces the same results ^2^
- **Objectivity:** Objective research can be defined as research that is: unbiased or impartial, value-free, reliable or trustworthy, or factual or real ^3^

**References:**

1. Frampton, G. K., Whaley, P., Bennett, M. G., Bilotta, G., Dorne, J., Eales, J., James, K., Kohl, C., Land, M., Livoreil, B., Makowski, D., Muchiri, E., Petrokofsky, G., Randall, N., & Schofield, K. A. (2022). Principles and framework for assessing the risk of bias for studies included in comparative quantitative environmental systematic reviews. *Environmental Evidence*, *11*(1). <https://doi.org/10.1186/s13750-022-00264-0>
2. *Research reliability*. (n.d.). Research-Methodology. <https://research-methodology.net/research-methodology/reliability-validity-and-repeatability/research-reliability/>
3. Resnik, D. (2001). Objectivity of Research: Ethical Aspects. In *Elsevier eBooks* (pp. 10789–10793). <https://doi.org/10.1016/b0-08-043076-7/00157-1>
